# The Honeybee Associate *Galleria mellonella* Can Acquire *Arsenophonus apicola* Through Oral and Parenteral Infection Routes

**DOI:** 10.1111/1462-2920.70088

**Published:** 2025-04-13

**Authors:** Trefor Simmons, Pol Nadal‐Jimenez, Gregory D. D. Hurst

**Affiliations:** ^1^ Institute of Infection, Veterinary and Ecological Sciences University of Liverpool Liverpool UK

**Keywords:** apiculture, infection, social insects, waxworm

## Abstract

Members of the genus *Arsenophonus* are classically considered to be vertically transmitted endosymbiotic associates of invertebrates. Acquisition of *Arsenophonus apicola* by *Apis mellifera* honeybees through social and environmental pathways raises the possibility that this species can infect a broader range of host species. In this study, we tested whether a natural inhabitant of bee hives, the wax moth *Galleria mellonella*, was a suitable host for *A. apicola*. We first demonstrated *A. apicola* colonised *G. mellonella* larvae following injection at doses as low as 10^4^ CFU. A similar capacity of *A. apicola* to infect *G. mellonella* orally was evidenced, impacting waxworm development and mortality. Microscopy indicated that *A. apicola* crossed from gut to hemocoel in the *G. mellonella* crop, inducing melanisation. PCR screening of *Galleria* individuals in an apiary sample confirmed exposure of *Galleria* in the hive context. We conclude that *A. apicola* is capable of infecting and damaging hive associates. These findings raise two onward avenues of research: first, to investigate whether *A. apicola*'s presence could protect hives against *Galleria* infestations, and second, to utilise model insect *G. mellonella* for immunity research to uncover the interplay between *A. apicola* and insect host defences whilst elucidating virulence factors utilised by *A. apicola* during infection.

## Graphical abstract

Created in BioRender.com *Galleria mellonella* larvae were exposed to *Arsenophonus apicola* orally and via injection, alongside negative control treatments. The exposed larvae were confirmed to be infected by monitoring GFP expression and exhibited increased mortality and melanisation, alongside delayed pupation.

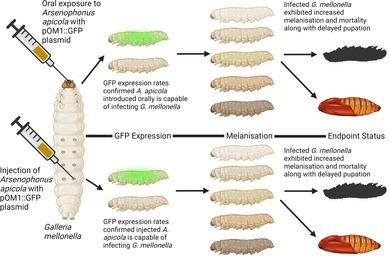

## 1 Introduction

*Arsenophonus* is a clade of insect‐associated bacteria within the gammaproteobacteria. Previous work on this genus established that most strains had exclusively vertical modes of transmission in their insect hosts. Across its distribution (estimated to be 5% of all arthropod species [Duron et al. 2008]), the clade notably includes vertically transmitted obligate symbionts in blood‐feeders (Dale et al. 2006), facultative symbionts that impact pesticide sensitivity (Cai et al. 2024) and male‐killers with mixed modes of transmission in parasitic wasps (Nadal‐Jimenez, Parratt et al. 2023).

To date, only one strain has been shown to transmit exclusively horizontally (Yañez et al. 2016; Drew et al. 2021). This strain, recently formally described as *Arsenophonus apicola* (Nadal‐Jimenez et al. 2022a), infects the honey bee *Apis mellifera* . Infection frequency shows seasonal dynamics in the UK and Ireland, with a pronounced peak in July–August (Drew et al. 2021; Almeida et al. 2023). Within this seasonal cycle, hives lose infection overwinter. *Arsenophonus apicola* is more commonly found in weakened hives and has been hypothesised to increase the susceptibility of bees to co‐infection, potentially leading to colony collapse (Budge et al. 2016). There is also emerging evidence of an anthropogenic impact on *A. apicola* infection patterns, with *A. mellifera* hives more likely to be infected with this bacterium in areas with intense agricultural use (Gorrochategui‐Ortega et al. 2022).

Bees can acquire *A. apicola* infection through social transfer within the hive (Drew et al. 2021). Whilst infection is spatially heterogeneous, the majority of worker bees in an infected hive typically test positive for *A. apicola* where it is present, the exception being newly emerged workers. Experiments indicate social transfer particularly follows from trophallaxis, although it can also be observed without direct feeding contact (Drew et al. 2021). This transmission mode is also reflected in its primary colonisation of the foregut of the bee (Corby‐Harris et al. 2014).

The capacity of *A. apicola* to transfer infectiously between *A. mellifera* individuals within the hive, between hives and from their environment suggests that this microbe may have a host range beyond *A. mellifera* itself. Insect species that are co‐resident within the hive represent particularly strong candidates for acquiring infection. One of the most common of these is the wax moth *Galleria mellonella*. Moth activity causes hive damage, particularly from the silk that can entangle emerging adult worker bees and can also vector destructive honey bee pathogens like black queen cell virus (Levitt et al. 2013; Traiyasut et al. 2016). Importantly for this study, *G*. *mellonella* is commonly found in weakened hives (Sohail et al. 2017), which are also considered particularly likely to carry *Arsenophonus* spp. (Kwadha et al. 2017; Budge et al. 2016). Spillover of *A. apicola* into *G*. *mellonella* would be important for two reasons. First, it may provide novel routes of infection and infection reservoirs for both parties. Second, spillover from *A. mellifera* to *G. mellonella* may alter the development of galleriasis (waxworm damage to infested hives). For instance, if wax moths acquire and are harmed by *A. apicola*, the impact of wax moth infestation could be ameliorated. If this hypothesis is confirmed, *A. apicola* infection in *A. mellifera* hives would represent a novel form of protective symbiosis.

In this study, we tested the capacity for *A. apicola* to establish in *G. mellonella* waxworms when introduced by injection or through feeding. Within this, we examine the dose dependency of infection, the impact of infection on host development and mortality, and the means of establishing hemocoel infections following an oral inoculum. Our results indicate that *G. mellonella* is a suitable host for *A. apicola* that can establish infections within the hemocoel following oral challenge.

## 2 Materials and Methods

### 2.1 Materials

*Arsenophonus apicola* was cultured on brain heart infusion agar media (BHI, Oxoid) at 30°C for 4 days before the transfer of individual colonies to liquid BHI medium (BHI, Oxoid) for further growth at 30°C under constant agitation for 24 h. The type strain ArsBeeUS^T^ (DSM113403^T^ = LMG 32504^T^ = CECT 30499^T^) was used, modified to carry a pOM1::GFP plasmid conferring spectinomycin resistance, providing ease of visualisation under epifluorescence (Nadal‐Jimenez et al. 2022b).

*Galleria mellonella* larvae were purchased from livefoodsdirect.co.uk and kept in sterilised plastic containers with sawdust and flapjack pieces as their sole food source (no water was provided) at 25°C and 50% relative humidity out of direct light. No larvae were observed to be affected by mould during incubation. For all trials, late instar larvae of similar size were selected over 20 mm body length and over 200 mg body weight without visible melanisation (Firacative et al. 2020). Selected larvae were fasted for 24 h prior to oral treatment.

### 2.2 Dose Response for *A. apicola* Infection in *G. mellonella* Following Injection

*A. apicola* was grown in BHI with spectinomycin for 4 days under aerobic conditions at 30°C. 2 μL of this culture was injected directly into *G*. *mellonella* larvae through the last left abdominal proleg (Ramarao et al. 2012). Dose response was investigated through concurrently introducing 10^−0^‐ to 10‐fold^−4^ dilutions and a BHI uninfected control (with added spectinomycin), in a total of 24 replicates (larvae injected) for each treatment. The original titre of introduced microbes was calculated *post hoc* through serial dilution on BHI agar under spectinomycin selection. Spectinomycin was shown to have no significant impact on larval health (reported in Supporting Information S1) when compared to the control cohort injected with spectinomycin‐free BHI under the same conditions described above.

The response variables measured daily for 7 days were: (i) The condition of the larvae, with any observed malaise of the insect or mortality; (ii) presence of clear GFP fluorescence indicating a high density of infecting *A. apicola*; (iii) host melanisation/nodulation response indicating a cellular immune response (Ratcliffe and Walters 1983). All observations are reported in Supporting Information S1. *A*. *apicola* proliferation was monitored in whole live insects, detected using a M165 FC Leica stereoscope equipped with a Leica EL6000 external light source for fluorescence excitation and visualised using the GFP plus filter [480/40 nm (460–500 nm); Leica Microsystems (UK) limited]. Waxworm melanisation patterns were graded on a scale adapted from (Kay et al. 2019) as 0 = unmelanised, 1 = evidence of nodulation, 2 = ‘lateral line’ melanisation, 3 = systemic melanisation covering more than 50% of the cuticle and 4 = complete melanisation. Examples of systemic fluorescence vs. autofluorescence and the different levels of melanisation can be found in Supporting Information S4.

### 2.3 Dose Response for *A. apicola* Infection *G. mellonella* Following Oral Exposure

The oral dose–response experiment was conducted under similar conditions to the injection assay above. The same quantity of *A. apicola* suspended in BHI (at various dilutions) with spectinomycin was introduced orally by gravage to *G*. *mellonella* waxworms according to the method used in (Ramarao et al. 2012) with 24 replicates/treatment. *G. mellonella* condition was then monitored daily over 8 days, observing the same response variables as the ones used in the injections and comparing them to a BHI uninfected control (with added spectinomycin). All observations are reported in Supporting Information S1. The original dose was confirmed using serial dilution plates as previously. Spectinomycin was shown to have no significant impact on larval health (reported in Supporting Information S1) when compared to the control cohort orally exposed to spectinomycin‐free BHI under the same conditions described above.

### 2.4 Development and Mortality Following Oral Challenge With *A. apicola*

To gain a more precise measure of the impact of *A. apicola* on *G. mellonella* development and mortality, a larger cohort of waxworms was subject to either oral exposure to live *A*. *apicola* (Aa) [2 μL of 54,000 CFU/μL] versus the same dose of heat‐killed *A. apicola* (AaX, culture incubated at 90^o^C for 10 min before administration) and unchallenged controls. *G*. *mellonella* were incubated at 25°C for 12 days and, at this endpoint, scored in terms of life stage reached (larva, pupa, adult) and mortality (live/dead). A total of 56 replicates per treatment were run.

### 2.5 Visualising Gut‐Haemolymph Transit of *A. apicola*

2.65 × 10^5^ CFU of *A*. *apicola* was introduced orally to *G. mellonella* using the method detailed previously alongside the same dose of heat‐killed *A. apicola*. Five days post feeding, intact *G. mellonella* guts were dissected and mounted whole, including the foregut/crop, midgut and hindgut. These were then cut open and flattened before fixation in 4% paraformaldehyde and subsequent washing with PBS. The material was then mounted in Vectashield (Vector labs H‐1200‐10) containing DAPI to enable host cell imaging as well as more detailed observation of bacterial localisation via GFP fluorescence. The orientation of the gut and the retention of the fluorophores post‐fixation were confirmed under an epifluorescent microscope before samples were imaged using an ANDOR Dragonfly 600 spinning disk confocal microscope.

### 2.6 PCR Screening of *Galleria* From an *A. apicola* Infected Apiary

A *Galleria*‐infested hive (53.0286269, −0.4733653) was identified in the summer of 2024 by a collaborator at the Lincolnshire Bee‐keepers Association. Three adult *A. mellifera* bees and *Galleria* at various life stages were collected and screened for the presence of *Arsenophonus* spp. following DNA extraction from the haemolymph of the different individuals using the methods described in (Senior and Titball 2021) and (Drew et al. 2021) and using primers Ars16SF: 5′GGGTTGTAAAGTACTTTCAGTCGT and Ars16SR: 5′CGCAGGCTCGCCTCTCTC under the following PCR conditions: initial denaturation (3 min at 94^o^C), 35 cycles of denaturation (15 s at 92^o^C) annealing (1 min at 59^o^C) and extension (30 s at 72°C) before a final extension (4 min at 72^o^C) based on previous screening protocols (Nadal‐Jimenez, Frost et al. 2023). DNA from pure cultures of *A. apicola* (ArsBeeUS^T^) and *E. coli* K‐12 (MG1655) was used as the positive and negative controls, respectively (extracted from colonies by boiling for 15 min in ddH_2_O). Samples were deemed positive for *Arsenophonus* upon evidence of an 820 bp amplicon that, when sequenced, matched *Arsenophonus* spp.

## 3 Statistical Analyses

### 3.1 Analysis of the Dose‐Dependent Response in *A. apicola*‐Infected *G. mellonella*

We interpreted the dose‐dependent response in *A. apicola‐*infected *G. mellonella* waxworms using time‐to‐event analysis for both injected and oral introduction (gravage) treatments. For analysing the dose‐dependent survival of infected larvae, the ‘event of interest’ (1) was defined as mortality and larvae were right censored (0) at the end of the trial if the individual was still alive. To analyse the dose‐dependent infection (confirmed via fluorescence) rate, the ‘event of interest’ (1) was defined as observable fluorescence and larvae were right censored (0) at the end of the trial if still uninfected. For analysing the dose‐dependent melanisation of infected larvae, the ‘event of interest’ (1) was defined as melanisation scoring ≥ 2 and larvae were right censored (0) at the end of the trial if still scored < 2. For analysing the dose‐dependent pupation rate of infected larvae, the ‘event of interest’ (1) was defined as pupation and larvae were right censored (0) at the end of the trial if still larvae.

In all cases, Kaplan–Meier survival curves were created using the ‘ggplot2’ package in R studio (RStudio Team 2020). Due to the limited range and intervals of observation, the ‘step‐downs’ in each curve were deemed too steep to lead to a productive estimation of median time‐to‐event (e.g., LT_50_). Overall heterogeneity between all doses for a treatment type was measured with likelihood ratio tests with the Cox Proportional Hazard model using the ‘survminer’ package in R studio. *Post hoc* testing for between‐dose significance and hazard ratio within a treatment type was performed by using pair‐wise likelihood ratio testing according to the Cox Proportional Hazard Model with Bonferroni corrected *α*‐values (dividing the original *α*‐value by the number of comparisons being made) between pairs of doses using the ‘survminer’ package in R studio. Code for analyses can be found in Supporting Information C.

### 3.2 Analysis of Development and Mortality Following Oral Challenge With *A. apicola*

We interpreted the outcome distribution after long‐term infection and incubation by describing each class of outcome as a proportion of the whole cohort and tested for heterogeneity by contingency Chi‐squared testing (6 treatments × 2 outcomes). In cases where the H_0_ of homogeneity was rejected, *post hoc* tests were performed on a pairwise basis, using Chi‐squared testing and Bonferroni correction of α value.

## 4 Results

### 4.1 Dose Response for *A. apicola* Infection in *G. mellonella* Following Injection

**FIGURE 1:**
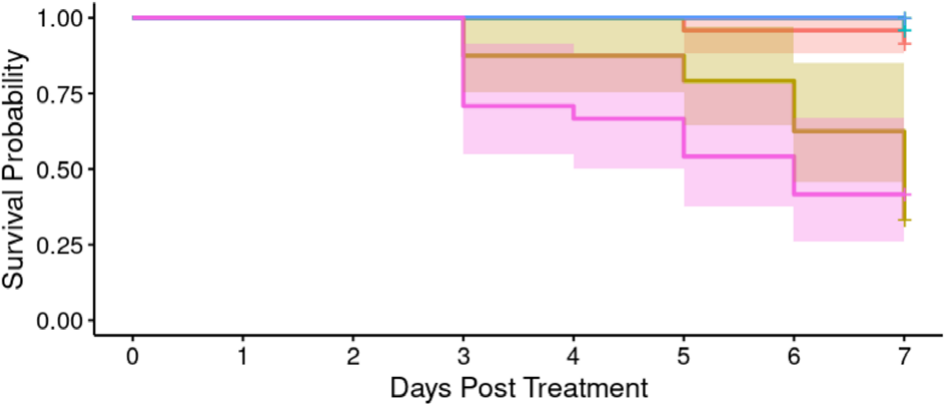
Survival probability over time of *G. mellonella* larvae infected with different concentrations of *A*. *apicola* pOM1::GFP introduced via injection (*n* = 24 replicates of each). Blue = control group (BHI without *A*. *apicola*); pink = original dose (1.2 × 10^6^ CFU); yellow = 10‐fold dilution (1.2 × 10^5^ CFU); teal = 100‐fold dilution (1.2 × 10^4^ CFU); red = 1000‐fold dilution (1.2 × 10^3^ CFU); green = 10,000‐fold dilution (1.2 × 10^2^ CFU). Coloured bands either side of the survival curve indicate the 95% confidence intervals for the Kaplan–Meier survival estimates.

*A. apicola* injected into *G*. *mellonella* haemolymph induced concentration‐dependent mortality over the 7 days following initial introduction (Figure 1). Mortality was observed where the initial bacterial load was either the undiluted culture (1.2 × 10^6^ CFU) or a 10‐fold dilution of this. Analysis indicated significant heterogeneity in survival between groups according to *A. apicola* dose (Cox proportional hazard LRT: χ^2^ = 69.87, 5 d.f., *p* < 0.01). *Post hoc* analysis comparing each dose to the BHI control indicated a significant difference in survivorship over time (in days) in the original 1.2 × 10^6^ CFU dose (Cox proportional hazard LRT: χ^2^ = 24.83, 1 d.f., *p* < 0.003; HR: exp.(coef) = 9.2 × 10^8^) and in the 10‐fold dilution (Cox proportional hazard LRT: χ^2^ = 29.87, 1d.f, *p* < 0.003; HR: exp.(coef) = 1 × 10^10^), but with no evidence of mortality increase in the 100‐, 1000‐ or 10,000‐fold dilution. The original dose of 1.2 × 10^6^ CFU and the 10‐fold dilution were both significantly different from the 100‐fold dilution (Supporting Information S2) but were not significantly different from each other. This analysis supports a threshold effect with outcome grouping of a ‘high dose’ and observed mortality (1.2 × 10^6^ CFU and 1.2 × 10^5^ CFU *A*. *apicola*) and a ‘low dose’ with no significant mortality (between 1.2 × 10^4^ CFU and 120 CFU *A*. *apicola*).

**FIGURE 2:**
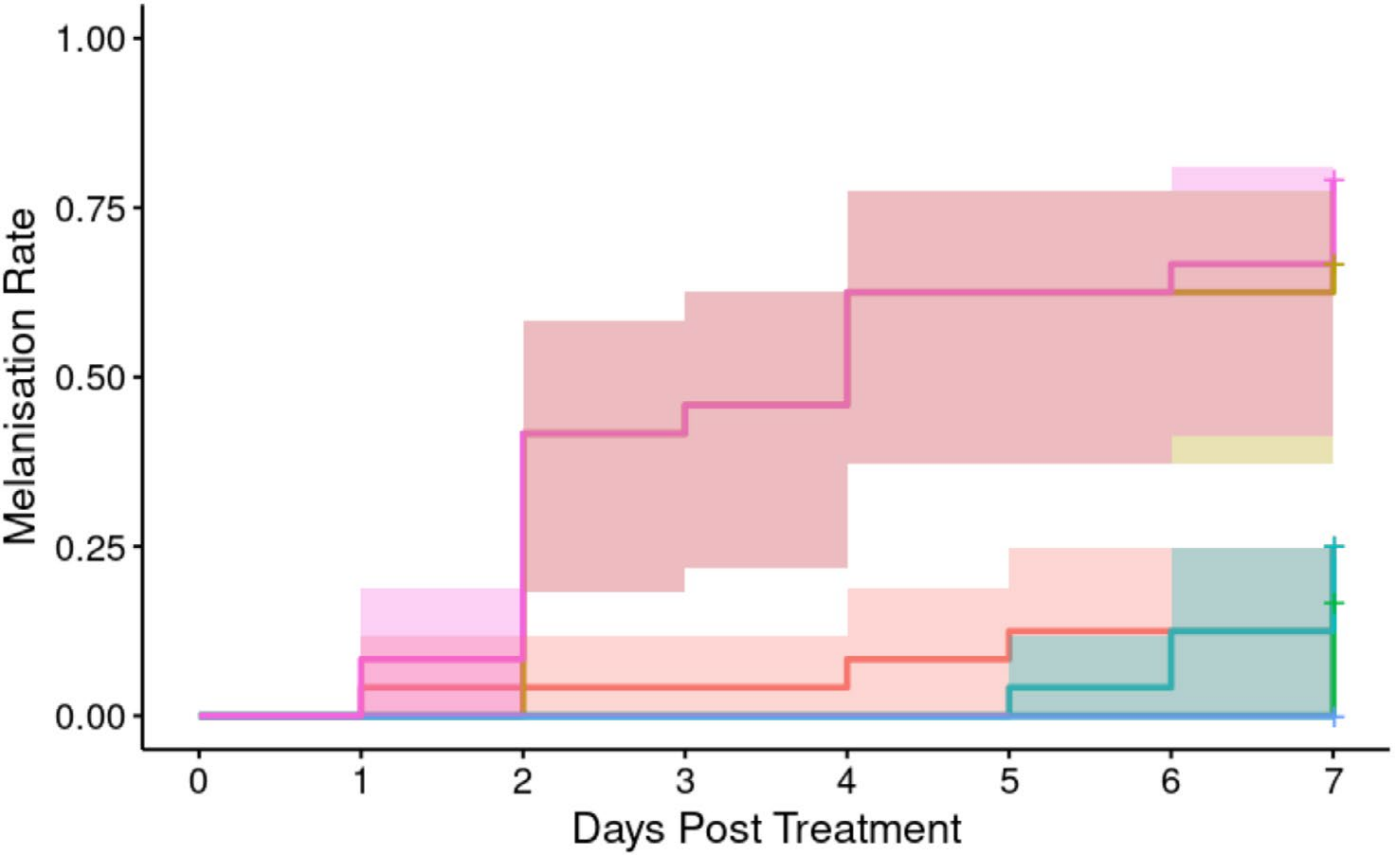
Proportion of larvae exhibiting melanisation (score ≥ 2) over time with different initial doses (*n* = 24 each) of *A*. *apicola* pOM1::GFP introduced via injection. Blue = control group (BHI without *A*. *apicola*); pink = original dose (1.2 × 10^6^ CFU); yellow = 10‐fold dilution (1.2 × 10^5^ CFU); teal = 100‐fold dilution (1.2 × 10^4^ CFU); red = 1000‐fold dilution (1.2 × 10^3^CFU); green = 10,000‐fold dilution (1.2 × 10^2^ CFU). Coloured bands either side of the survival curve indicate the 95% confidence intervals for the Kaplan–Meier survival estimates.

*Arsenophonus apicola* injected into the *G*. *mellonella* hemolymph induced concentration‐dependent melanisation over the 7 days following initial introduction (Figure 2; Table 1). Progressive melanisation (score ≥ 2 as per Supporting Information S4) was observed, evidencing a haemocoel immune response, where the initial bacterial load was either undiluted culture (1.2 × 10^6^ CFU) or a 10‐fold serial dilution of this. Overall analysis of progression to melanisation score ≥ 2 indicated significant heterogeneity in the melanisation rate between groups (Cox proportional hazard LRT: χ^2^ = 63.58, 5 d.f., *p* < 0.01). *Post hoc* analysis comparing to BHI control indicated a significant difference in melanisation over time (in days) in all doses used (Supporting Information S2).

**TABLE 1:** Melanisation index over time of *G. mellonella* infected with different concentrations of *A*. *apicola* via injection.

Treatment	Days post treatment						
	1	2	3	4	5	6	7
Original dose	0.38 (24)	1.25 (24)	1.50 (24)	2.21 (24)	2.21 (24)	2.33 (24)	2.75 (24)
10^−1^	0.21 (24)	1.08 (24)	1.63 (24)	2.29 (24)	2.42 (24)	2.57 (23)	2.96 (23)
10^−2^	0.21 (24)	0.38 (24)	0.38 (24)	0.54 (24)	0.61 (23)	0.78 (23)	1.18 (17)
10^−3^	0.21 (24)	0.33 (24)	0.33 (24)	0.46 (24)	0.58 (24)	0.59 (22)	1.11 (18)
10^−4^	0.21 (24)	0.21 (24)	0.21 (24)	0.38 (24)	0.48 (21)	0.39 (18)	1.09 (11)
BHI control	0.25 (24)	0.25 (24)	0.25 (24)	0.25 (24)	0.35 (23)	0.32 (19)	0.40 (10)

*Note:* Values given are the mean score over larval replicates (using the scale adapted from with sample size in parentheses); individuals that pupated during the trial were no longer scored (dead individuals were retained).

**FIGURE 3:**
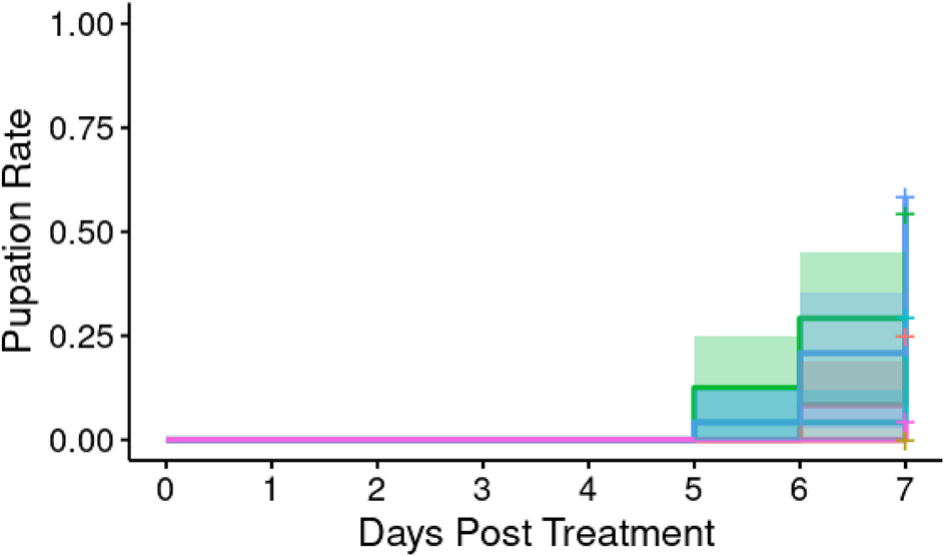
Proportion of larvae pupating over time with different initial doses (*n* = 24 each) of *A*. *apicola* pOM1::GFP introduced via injection. Blue = control group (BHI without *A*. *apicola*); pink = original dose (1.2 x10^6^ CFU); yellow = 10‐fold dilution (1.2 × 10^5^ CFU); teal = 100‐fold dilution (1.2 × 10^4^ CFU); red = 1000‐fold dilution (1.2 × 10^3^ CFU); green = 10,000‐fold dilution (1.2 × 10^2^ CFU). Coloured bands either side of the survival curve indicate the 95% confidence intervals for the Kaplan–Meier survival estimates.

*A. apicola* injected into the *G*. *mellonella* haemolymph likewise induced concentration‐dependent pupation delay over the 7 days following initial introduction (Figure 3). Pupation delay was observed where the initial bacterial load was either the undiluted 1.2 × 10^6^ CFU or a 10‐fold dilution of this dose. Overall analysis indicated significant heterogeneity in the pupation rate between groups (Cox proportional hazard LRT: χ^2^ = 42.57, 5 d.f., *p* < 0.01). *Post hoc* analysis comparing to BHI control indicated a significant difference in the pupation rate over time (in days) in the original dose (Cox proportional hazard LRT: χ^2^ = 18.48, 1 d.f., *p* < 0.03; HR: exp.(coef) = 0.04) and the 10‐fold dilution (Cox proportional hazard LRT: χ^2^ = 24.83, 1 d.f., *p* < 0.03; HR: exp.(coef) = 4.84 × 10^−2^). There was no significant difference between these two doses (Supporting Information S2), and both were shown to differ significantly from the 100‐fold dilution and all lower doses.

**FIGURE 4:**
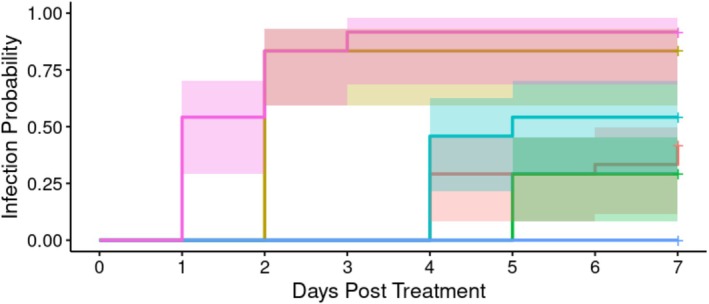
Proportion of larvae exhibiting systemic GFP expression over time with different initial doses (*n* = 24 each) of *A*. *apicola* pOM1::GFP introduced via injection. Blue = control group (BHI without *A*. *apicola*); pink = original dose (1.2 × 10^6^ CFU); yellow = 10‐fold dilution (1.2 × 10^5^ CFU); teal = 100‐fold dilution (1.2 × 10^4^ CFU); red = 1000‐fold dilution (1.2 × 10^3^ CFU); green = 10,000‐fold dilution (1.2 × 10^2^ CFU). Coloured bands either side of the survival curve indicate the 95% confidence intervals for the Kaplan–Meier survival estimates.

*A. apicola* injected into the *G*. *mellonella* haemolymph induced concentration‐dependent systemic infection (defined as presence of GFP fluorescence throughout the larva) over the 7 days following initial introduction (Figure 4). Systemic infection was established at all of the dilution levels but was most commonly observed where the initial bacterial load was either the undiluted culture (1.2 × 10^6^ CFU) or a 10‐fold dilution of this. Overall analysis indicated significant heterogeneity in the systemic infection rate between groups (Cox proportional hazard LRT: χ^2^ = 103.6, 5 d.f., *p* < 0.01). *Post hoc* analysis comparing to BHI control indicated a significant difference in the infection rate over time (in days) in all doses used (Supporting Information S2).

For the cohorts injected with either the BHI control or a dose of *Arsenophonus apicola*, the GFP signal, melanisation and survival were all analysed as positive predictor values (PPVs) for each other, where one is viewed as a ‘test’ value predicting the ‘condition’ value and used to calculate PPV as the proportion of ‘true positives’ to ‘all positives’. All individuals that died during the 7 days of observation were also positive for GFP signal and melanisation. Endpoint melanisation was shown to predict mortality (PPV_mel_ = 0.65) slightly better than endpoint GFP signal (PPV_GFP_ = 0.46). Endpoint melanisation was shown to predict endpoint GFP condition (PPV_mel_ = 1) more reliably than endpoint GFP predicted endpoint melanisation (PPV_GFP_ = 0.71). These PPVs (along with the timing data) suggest that most *Galleria* larvae injected with the *Arsenophonus apicola* exhibit an ordered infection phenotype progressing from melanisation to GFP‐evidenced systemic infection to mortality.

### 4.2 Dose Response for *A. apicola* Infection in *G. mellonella* Following Oral Exposure

**FIGURE 5:**
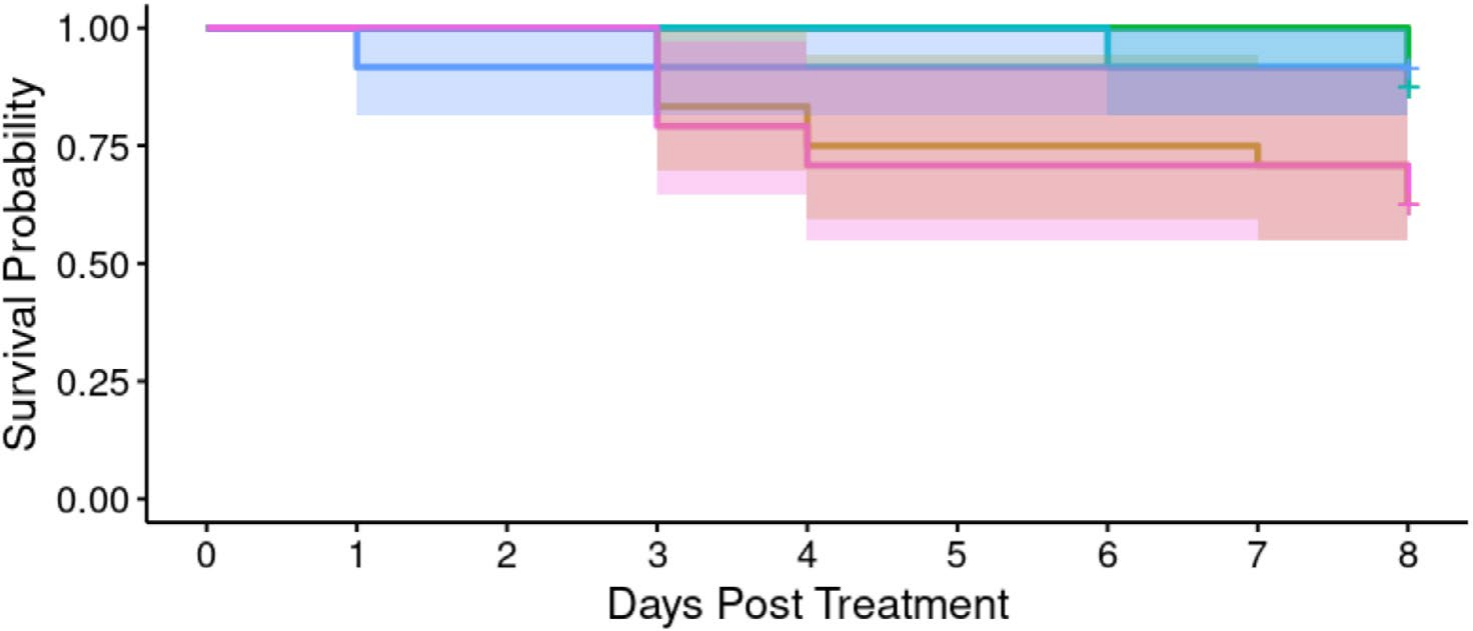
Survival probability over time of *G. mellonella* larvae infected with different concentrations (*n*‐24 each) of *A*. *apicola* pOM1::GFP introduced orally. Blue = control group; pink = original dose (1.1 × 10^6^ CFU); yellow = 10‐fold dilution (1.1 × 10^5^ CFU); teal = 100‐fold dilution (1.1 × 10^4^ CFU); red = 1000‐fold dilution (1.1 × 10^3^ CFU); green = 10,000‐fold dilution (1.1 × 10^2^ CFU). Coloured bands either side of the survival curve indicate the 95% confidence intervals for the Kaplan–Meier survival estimates.

*Arsenophonus apicola* introduced to the *G*. *mellonella* digestive tract via oral exposure was observed to induce concentration‐dependent mortality over the 8 days following initial introduction (Figure 5). Mortality was observed where the initial bacterial load was either the undiluted 1.1 × 10^6^ CFU or a 10‐fold dilution of this concentration. Overall analysis indicated significant heterogeneity in survival between groups (Cox proportional hazard LRT: χ^2^ = 13.84, 5 d.f., *p* = 0.02). *Post hoc* analysis comparing to BHI control indicated a difference in survivorship over time (in days) in the original 1.1 × 10^6^ CFU dose (Cox proportional hazard LRT: χ^2^ = 5.42, 1 d.f., *p* = 0.02; HR: exp.(coef) = 4.90) and in the 10‐fold dilution (Cox proportional hazard LRT: χ^2^ = 5.42, 1 d.f., *p* = 0.02; HR: exp.(coef) = 4.90), but with no evidence of mortality increase in the 100, 1000 or 10,000‐fold dilution (see also Supporting Information S2).

**FIGURE 6:**
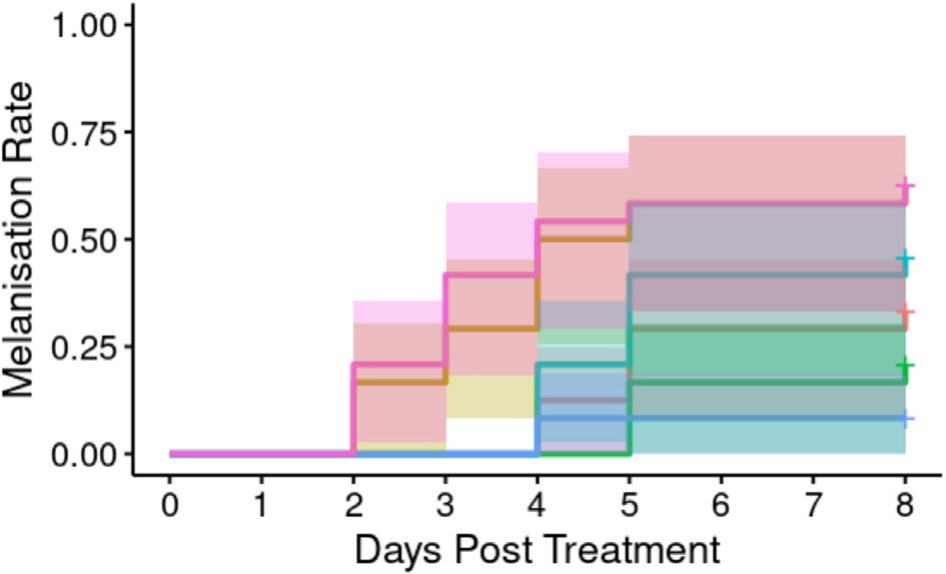
Proportion of larvae exhibiting melanisation (score ≥ 2) over time with different initial doses (*n* = 24 each) of *A*. *apicola* pOM1::GFP introduced orally. Blue = control group (BHI without *A*. *apicola*); pink = original dose (1.2 × 10^6^ CFU); yellow = 10‐fold dilution (1.2 × 10^5^ CFU); teal = 100‐fold dilution (1.2 × 10^4^ CFU); red = 1000‐fold dilution (1.2 × 10^3^ CFU); green = 10,000‐fold dilution (1.2 × 10^2^ CFU). Coloured bands either side of the survival curve indicate the 95% confidence intervals for the Kaplan–Meier survival estimates.

*A. apicola* introduced into the *G*. *mellonella* digestive tract induced concentration‐dependent melanisation of the waxworm over the 8 days following initial introduction (Figure 6; Table 2). Progressively increasing melanisation (score ≥ 2, see methods) was observed, evidencing haemocoel immune response, where the initial bacterial load was either the undiluted 1.1 × 10^6^ CFU or a 10‐fold serial dilution of this. Overall analysis indicated significant heterogeneity in the melanisation rate between groups as measured at the endpoint (Cox proportional hazard LRT: χ^2^ = 31.69, 5 d.f., *p* < 0.01). *Post hoc* analysis comparing to BHI control indicated a significant difference in the melanisation rate over time (in days) in all doses used (Supporting Information B), with the exception of the 10,000‐fold dilution.

**TABLE 2:** Melanisation index over time of *G. mellonella* infected orally with different concentrations of *A. apicola*.

Treatment	Days post treatment							
	1	2	3	4	5	6	7	8
Original dose	0.00 (24)	0.54 (24)	1.42 (24)	1.83 (24)	1.92 (24)	1.96 (23)	2.21 (19)	2.17 (18)
10^−1^	0.00 (24)	0.42 (24)	1.29 (24)	2.00 (24)	2.08 (24)	2.53 (19)	2.75 (16)	2.75 (16)
10^−2^	0.00 (24)	0.00 (24)	0.00 (24)	1.13 (24)	1.48 (23)	1.60 (20)	2.13 (15)	2.13 (15)
10^−3^	0.00 (24)	0.00 (24)	0.00 (24)	0.67 (24)	0.95 (22)	1.16 (19)	1.35 (17)	1.25 (16)
10^−4^	0.00 (24)	0.00 (24)	0.00 (24)	0.58 (24)	0.88 (24)	1.33 (18)	1.60 (15)	1.46 (13)
BHI control	0.33 (24)	0.33 (24)	0.33 (24)	0.63 (24)	0.64 (22)	0.80 (15)	1.09 (11)	1.22 (9)

*Note:* Values given are the mean score over larval replicates (using the scale adapted from with sample size in parentheses); individuals that pupated during the trial were no longer scored (dead individuals were retained).

**FIGURE 7:**
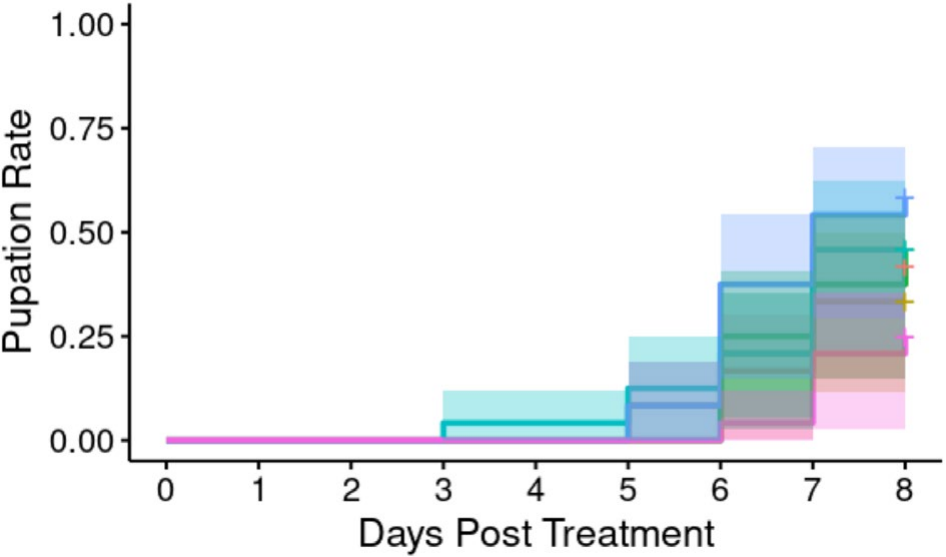
Proportion of larvae pupating following treatment with different concentrations (*n* = 24 each) of *A*. *apicola* pOM1::GFP introduced orally. Blue = control group; pink = original dose (1.1 × 10^6^ CFU); yellow = 10‐fold dilution (1.1 × 10^5^ CFU); teal = 100‐fold dilution (1.1 × 10^4^ CFU); red = 1000‐fold dilution (1.1 × 10^3^ CFU); green = 10,000‐fold dilution (1.1 × 10^2^ CFU). Coloured bands either side of the survival curve indicate the 95% confidence intervals for the Kaplan–Meier survival estimates.

We next analysed whether orally introduced *A*. *apicola* induced concentration‐dependent pupation delay over the 8 days following initial introduction (Figure 7). Pupation delay was observed where initial bacterial load was either the undiluted 1.2 × 10^6^ CFU or a 10‐fold dilution of this. Overall analysis indicated no significant heterogeneity in the pupation rate between groups (Cox proportional hazard LRT: χ^2^ = 7.53, 5 d.f., *p* = 0.2).

**FIGURE 8:**
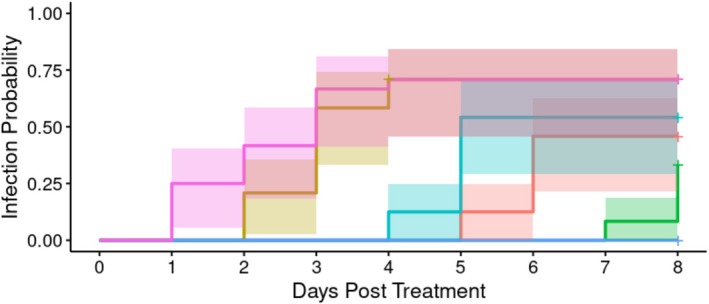
Proportion of larvae infected with different concentrations (*n*‐24 each) of *A*. *apicola* pOM1::GFP introduced orally. Blue = control group; pink = original dose (1.1 × 10^6^ CFU); yellow = 10‐fold dilution (1. × 10^5^ CFU); teal = 100‐fold dilution (1.1 × 10^4^ CFU); red = 1000‐fold dilution (1.1 × 10^3^ CFU); green = 10,000‐fold dilution (1.1 × 10^2^ CFU). Coloured bands either side of the survival curve indicate the 95% confidence intervals for the Kaplan–Meier survival estimates.

Orally introduced *A*. *apicola* induced concentration‐dependent systemic infection over the 8 days following initial introduction (Figure 8). Fluorescence was observed, evidencing disseminated infection, at all initial doses. The proportion of individuals showing systemic infection was highest where the initial bacterial load was either the undiluted dose (1.2 × 10^6^ CFU) or a 10‐fold dilution of this. Overall analysis indicated significant heterogeneity in the infection rate between groups (Cox proportional hazard LRT: χ^2^ = 59.08, 5 d.f., *p* < 0.01). *Post hoc* analysis comparing to the BHI control indicated a significant heterogeneity in the infection rate over time (in days) in all doses used (Supporting Information B).

For the cohorts treated with orally introduced BHI control or a dose of *Arsenophonus apicola*, the GFP signal, melanisation and survival were all analysed as PPVs for each other, where one is viewed as a ‘test’ value predicting the ‘condition’ value and used to calculate PPV as the proportion of ‘true positives’ to ‘all positives’. Nearly all individuals that died during the 7 days of observation were also positive for GFP signal and melanisation, except for 2 in the control group that died shortly after exhibiting melanisation (but no GFP signal). Endpoint melanisation was shown to predict mortality (PPV_mel_ = 0.52) slightly better than endpoint GFP signal (PPV_GFP_ = 0.40). Endpoint Melanisation was shown to predict endpoint GFP condition (PPV_mel_ = 0.96) more reliably than endpoint GFP‐predicted endpoint melanisation (PPV_GFP_ = 0.81). These PPVs (along with the timing data) suggest that most *Galleria* larvae treated with orally introduced *Arsenophonus apicola* exhibit an ordered infection phenotype progressing from melanisation to GFP‐evidenced systemic infection to mortality.

### 4.3 Development and Mortality Following Oral Challenge With *A. apicola*

The oral infection experiment was repeated with a single concentration of *A. apicola* [1.08 × 10^6^ CFU] compared to heat‐killed and no microbe control, examining the outcome at day 12 post inoculation (Table 3). Waxworm mortality was highest following exposure to live *A. apicola* (live Aa = 58.9% mortality; dead Aa = 19.6%; no treatment 3.6%; all *N* = 56). The null hypothesis of no heterogeneity in mortality associated with treatment was rejected (χ^2^ = 45.68, 2 d.f., *p* < 0.00001). *Post hoc* analysis indicates live *A. apicola* differed significantly from both the heat‐killed titre (χ^2^ = 18.11, 1 d.f., *p* < 0.01) and the unchallenged control groups (χ^2^ = 39.94, 1 d.f., *p* < 0.01).

**TABLE 3:** Outcome distribution of *G*. *mellonella* following live or heat‐treated oral infection.

	Live			Dead		Death censored data		
	Larva	Pupa	Adult	Larva	Pupa	% mortality	% pupation	% eclosion
Live *A. apicola*	3	5	15	16	17	58.9 (33/56)	86.9 (20/23)	65.2 (15/23)
Dead *A. apicola*	2	1	42	10	1	19.6 (11/56)	95.6 (43/45)	93.3 (42/45)
No manipulation control	0	4	50	2	0	3.6 (2/56)	100 (54/54)	92.6 (50/54)

*Note:* Following 12 days incubation at 25^o^C, the cohorts (*n* = 56) were scored for life stage (larva, pupa, adult) and mortality (live/dead). No dead adults were observed. Pupa and adult counts contributed to the pupation rate, whilst adult counts contributed to the eclosion rate. Death censoring was done by adjusting the *n*‐values to include only survivors at the endpoint of incubation. All percentages are given with cohort proportions presented in brackets.

Based on the life stage data, development appeared to be slowed by *A. apicola*, as evidenced by decreased rates of pupation and eclosion in the live *A. apicola* pOM1::GFP treated group. However, when dead individuals were removed from the analysis, the pupation rate of the live‐bacteria‐fed group was not significantly different from that of the heat‐killed titre (χ^2^ = 1.65, 1 d.f., *p* = 0.2) or the unchallenged control groups (χ^2^ = 2.21, 1 d.f., *p* = 0.14). The death censored data did detect a significant difference in the eclosion rate between the live dose and both the heat‐killed treatment (χ^2^ = 8.87, 1 d.f., *p* < 0.01) and the unchallenged control groups (χ^2^ = 9.19, 1 d.f., *p* < 0.01). The significant increase in mortality between the heat‐killed titre and the unchallenged control groups (χ^2^ = 7.04, 1 d.f., *p* < 0.01) was not reflected in a significant difference in the pupation rate of the surviving individuals (χ^2^ = 0.03, 1 d.f., *p* = 0.86) or eclosion rate (χ^2^ = 0.02, 1 d.f., *p* = 0.88).

### 4.4 Visualising Gut‐Haemolymph Transit of *A. apicola*

**FIGURE 9:**
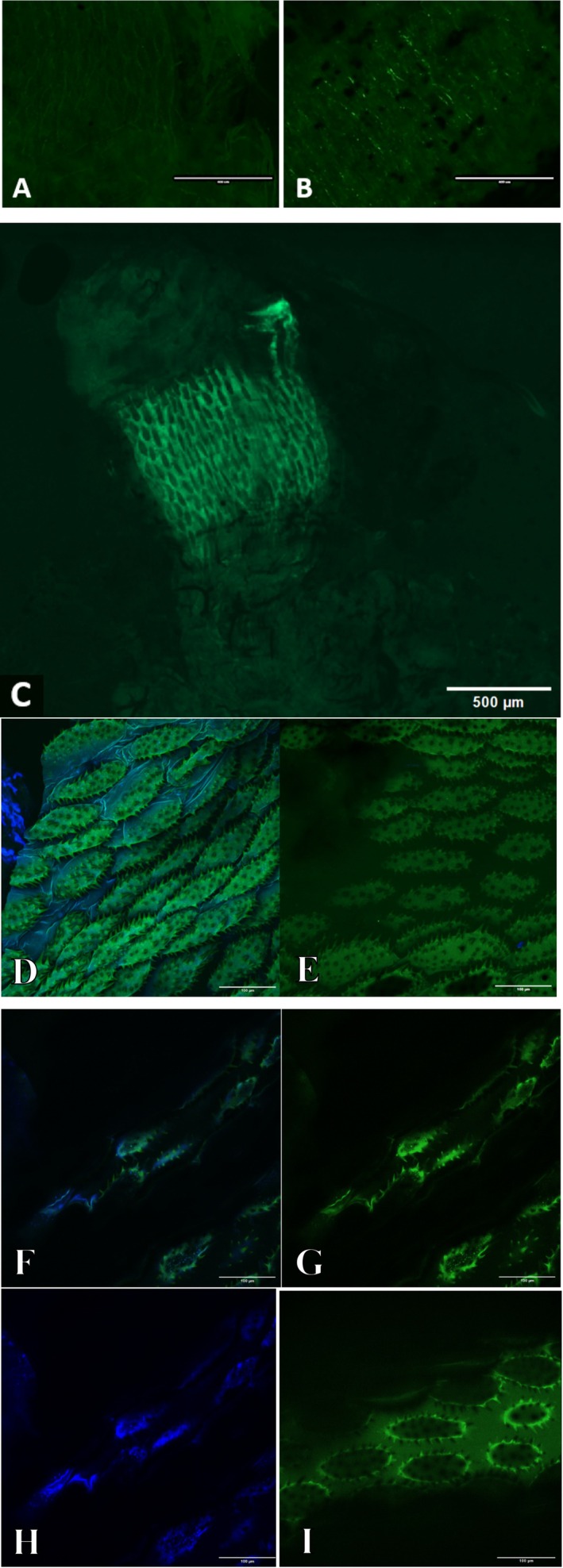
Epifluorescent microscopy images of *A. apicola*‐infected *G. mellonella* larvae. Disected tissue imaged: (A) control, section of foregut following introduction of heat‐killed *A. apicola*; (B) section of foregut following live *A. apicola* introduction, showing epifluoresence and melanisation; (C) example of a case of very high signal GFP epifluoresence following live *A. apicola* introduction. (D) z‐Stack of the foregut section from infected individual with GFP (green) and Dapi (blue) signals. (E) z‐Stack of the foregut section from control‐treated individual with GFP (green) and Dapi (blue) signals. (F) z‐Slice of the foregut section of infected individual showing combined GFP (green) [G] and Dapi (blue) (H) signals. (I) z‐Slice of the foregut section of control‐treated individual showing combined GFP (green) and Dapi (blue) signals. Scale bars A and B represent 400 μm, C represents 500 μm and D–I represent 100 μm.

Dissected gut tissue shows a consistent link between GFP expression from the *A. apicola* infection and surface melanisation that is pronounced in the foregut region (Figure 9). Compared to negative foreguts (imaged 5 days post treatment with heat‐killed *A. apicola*), in infected larvae, small nodules of melanised tissue can be observed on the surface as well as the GFP expression indicative of bacterial growth.

### 4.5 Screening *Galleria* From an *Arsenophonus‐*Infected Apiary

The infection status of samples from a single *Galleria‐infested* hive was assessed by *Arsenophonus‐*specific PCR screening of individual haemolymph extracts. *Arsenophonus* infection was present in all three honeybees tested, indicating that the infection was present in this apiary. *Galleria* taken from the apiary tested positive, but at a lower rate (23% of larvae and 25% of pupae testing positive). The single adult moth collected tested negative for *Arsenophonus* (Table 4). BLAST comparison of sequenced amplicons verified all PCR‐positive amplicons as *A. apicola*.

**TABLE 4:** *Arsenophonus* screening assay results for *G. mellonella* individuals of different life stages derived from a Lincolnshire apiary.

Species	*Galleria mellonella*		
Life stage	Larva	Pupa	Adult
*A. apicola* Positive	3	2	0
*A. apicola* Negative	10	6	1

## 5 Discussion

The clade *Arsenophonus* contains a broad range of insect vertically transmitted symbionts, including son‐killer parasites of *Nasonia* wasps (Duron et al. 2008) and coevolving nutritional mutualists of *Pseudolynchia* louse flies (Dale et al. 2006). *Arsenophonus apico*l*a* is unique within this bacterial clade, as it exhibits no evidence of vertical transmission between generations, seeming to rely solely on horizontal transmission between individuals (Drew et al. 2021). This evidence of intraspecific horizontal transfer forms the foundation of the hypothesis that *A*. *apicola* could ‘spillover’ and infect a broader range of insects, such as non‐hymenopteran hive mates like *G*. *mellonella* waxmoths (Nanetti et al. 2021). We investigated the potential for interspecific transfer using laboratory experiments, where *A. apicola* at different doses was introduced to waxworm larvae by injection and oral feeding, and the fate of infection was followed in terms of progression of infection, host development, host immune response (melanisation) and mortality.

Our experiments collectively demonstrate that *A. apicola* can infect and propagate in *G*. *mellonella* larvae following both injection and oral exposure. At high doses, *A. apicola* delayed development, was associated with host melanisation responses and also had a significant mortality impact. These data support *A. apicola* being able to colonise host species from different insect orders and that (at high doses) this colonisation has morbidity and mortality outcomes. It is notable that *A. apicola* was able to cross the gut–haemolymph barrier, which we observed occurring in the crop. This pattern of crop infection recapitulates the crop infection seen for *A. apicola* in *A. mellifera* (Drew et al. 2021), indicating that this may be a general infection process for *A. apicola*.

The scope and application of these conclusions are limited by our use of cultured inoculum: whilst effective for dose–response estimation, this process does not necessarily replicate the field‐realistic introduction of *A. apicola* to *G. mellonella* (most likely as an ingested contaminant of occupied *A. mellifera* hive material). The capacity to establish systemic infection from the lowest dose in our study (c. 100 CFU) supports the hypothesis that these *Arsenophonus* infections are prone to be acquired from environmental sources. Our data from a single hive suffering galleriasis indicate that the waxworms within hives where honeybees carry *Arsenophonus* are also exposed to and may become infected by this bacterium. One feature that may predispose *G. mellonella* to *A. apicola* infection is that both parties are more likely to be present in weakened *A. mellifera* hives (Kwadha et al. 2017; Budge et al. 2016).

Galleriasis—the infestation of hives with wax moths—can cause the loss of hive productivity and ultimately hives. Our findings indicate the potential for an unusual dynamic—that Galleriasis might be mitigated in its impact where the hive has *A. apicola*. This process would require *Galleria* to acquire *A. apicola* in honeybee hives and then moth development and survival to be impacted by the *A. apicola* infection. This potential relationship would make *A. apicola* a ‘protective symbiont’ at hive level. This possible impact in the hive environment represents an exciting avenue for onward research. The potential of *Arsenophonus apicola* as a treatment for acute galleriasis could be investigated, mediated by the need for evidence that it does not harm the honeybee host (or wider pollinator community) in the same way. More broadly, the case for the function of *Arsenophonus* as a protective symbiont would be strengthened by evidence of its specific pathogenicity in comparison with other transient and commensal bacteria common in honeybees and their hives, and indications that pathogenicity can occur consequent on the exposure level that occurs within hives.

Whilst not the main goal of the study, our results indicate that *G. mellonella* is a valid model system for onward research into the mechanism of the invasion and immune response associated with *A. apicola* infection. This use has three advantages. First, *G. mellonella* is much easier to maintain in the lab than *A. mellifera* (Schilcher et al. 2021), whose lab use is limited to worker bees isolated from hives. Second, laboratory use enables investigation with *A. apicola* strains expressing transgenic GFP or other genetic modifications that are not possible in the field. Third, there is a historical base of knowledge concerning the *G. mellonella* immune response (Browne et al. 2014; Ménard et al. 2021; Pereira et al. 2018; Trevijano‐Contador and Zaragoza 2019), together with the ease of haemolymph extraction in this host (Senior and Titball 2021), making antimicrobial peptide (AMP) activation studies more accessible than in other model systems.

## Author Contributions

**Trefor Simmons:** conceptualization, data curation, formal analysis, funding acquisition, investigation, methodology, visualization, writing – original draft, writing – review and editing. **Pol Nadal‐Jimenez:** conceptualization, investigation, methodology, resources, supervision, writing – review and editing. **Gregory D. D. Hurst:** conceptualization, formal analysis, funding acquisition, methodology, project administration, supervision, writing – original draft, writing – review and editing.

## Ethics Statement

The authors have nothing to report.

## Conflicts of Interest

The authors declare no conflicts of interest.

## Supporting information


Data S1.

Data S2.

Data S3.

Data S4.

Data S5.

## Data Availability

Data are publicly available on figshare. Raw data available at https://figshare.com/s/8893698f387c510730a8. The DOI's are listed below: Simmons, Trefor; Hurst, Greg; Nadal‐Jimenez, Pol (2025). Supporting Information S4; Melanisation and Flourescence Index Photos. figshare. Figure. https://doi.org/10.6084/m9.figshare.28554512.v1. Simmons, Trefor; Hurst, Greg; Nadal‐Jimenez, Pol (2025). Supporting Information S1; Observation Records.xlsx. figshare. Dataset. https://doi.org/10.6084/m9.figshare.25895650.v1. Simmons, Trefor; Hurst, Greg; Nadal‐Jimenez, Pol (2025). Supporting Information S2; Post hoc Tables.docx. figshare. Dataset. https://doi.org/10.6084/m9.figshare.25895653.v1. Simmons, Trefor; Hurst, Greg; Nadal‐Jimenez, Pol (2025). Supporting Information S3; Code used for Stats Analysis. R. figshare. Software. https://doi.org/10.6084/m9.figshare.25895641.v1. Simmons, Trefor; Hurst, Greg; Nadal‐Jimenez, Pol (2025). README.txt. S5 figshare. Online resource. https://doi.org/10.6084/m9.figshare.25895638.v1.
