# Supplementary material for: The Honeybee Associate Galleria mellonella Can Acquire Arsenophonus apicola Through Oral and Parenteral Infection Routes
Source: Environ Microbiol. 2025 Apr 13;27(4):e70088. doi: 10.1111/1462-2920.70088 (PMC11994876; doi:10.1111/1462-2920.70088)
Supplement: Supplementary file 2 — Data S2. [file EMI-27-e70088-s005.docx]

***Post-Hoc Pairwise Likelihood Ratio (Cox Proportional Hazard Model) Testing Tables***

Outline

The dose-dependent response in *A. apicola-*infected *G. mellonella* waxworms was interpreted using time-to-event analysis for both injected and force-fed treatments. For analysing the dose-dependent survival of infected larvae the “event of interest” (1) was defined as mortality and larvae were right censored (0) at the end of the trial if still alive. To analyse the dose-dependent infection (confirmed via fluorescence) rate the "event of interest” (1) was defined as observable fluorescence and larvae were right censored (0) at the end of the trial if still uninfected. For analysing the dose-dependent melanisation of infected larvae the “event of interest” (1) was defined as melanisation scoring ≥2 and larvae were right censored (0) at the end of the trial if still scored <2. For analysing the dose-dependent pupation rate of infected larvae the “event of interest” (1) was defined as pupation and larvae were right censored (0) at the end of the trial if still larvae.

*Post-hoc* testing for between-dose significance and hazard ratio within a treatment type was performed by using pair-wise likelihood ratio testing according to the Cox Proportional Hazard Model with Bonferroni corrected α-values (dividing the original α-value by the number of comparisons being made) between pairs of doses using the “survminer” package in R studio.

In all cases, the original critical value of 0.05 was divided by the number of pair-wise comparisons (15) to give the Bonferroni adjusted α-value: 0.003. Pairs which had heterogeneity shown to be significant at this level are presented with an asterisk e.g. 19.1 on 1 df, p=1e-05*.

Injected Dose Pair-wise Heterogeneity Tables

|  | **1.2x10^5^CFU** | **1.2x10^4^CFU** | **1.2x10^3^CFU** | **1.2x10^2^CFU** | **BHI control** |
| --- | --- | --- | --- | --- | --- |
| **1.2x10^6^CFU** | 0.05 on 1 df, p=0.8 | 19.1 on 1 df, p=1e-05* | 15.27 on 1 df, p=9e-05* | 24.83 on 1 df, p=6e-07* | 24.83 on 1 df, p=6e-07* |
| **1.2x10^5^CFU** | x | 23.41 on 1 df, p=1e-06* | 18.6 on 1 df, p=2e-05* | 29.87 on 1 df, p=5e-08* | 29.87 on 1 df, p=5e-08* |
| **1.2x10^4^CFU** | x | x | 0.37 on 1 df, p=0.5 | 1.39 on 1 df, p=0.2 | 1.39 on 1 df, p=0.2 |
| **1.2x10^3^CFU** | x | x | x | 2.82 on 1 df, p=0.09 | 2.82 on 1 df, p=0.09 |
| **1.2x10^2^CFU** | x | x | x | x | 0 on 0 df, p=1 |

Table 1: The results of pairwise likelihood ratio testing using Cox Proportional Hazard model on survival time-to-event data from the *Galleria* waxworms challenged with injected *Arsenophonus apicola* pOM1::GFP at different doses.

|  | **1.2x10^5^CFU** | **1.2x10^4^CFU** | **1.2x10^3^CFU** | **1.2x10^2^CFU** | **BHI control** |
| --- | --- | --- | --- | --- | --- |
| **1.2x10^6^CFU** | χ2=3.34 on 1 df, p=0.07 | χ2=25.45 on 1 df, p=5e-07* | χ2=29.15 on 1 df, p=7e-08* | χ2=33.23 on 1 df, p=8e-09* | χ2=50.64 on 1 df, p=1e-12* |
| **1.2x10^5^CFU** | x | χ2=14.92 on 1 df, p=1e-04* | χ2=18.66 on 1 df, p=2e-05* | χ2=22.97 on 1 df, p=2e-06* | χ2=42.36 on 1 df, p=8e-11* |
| **1.2x10^4^CFU** | x | x | χ2=1.01 on 1 df, p=0.3 | χ2=4.84 on 1 df, p=0.03 | χ2=22.51 on 1 df, p=2e-06* |
| **1.2x10^3^CFU** | x | x | x | χ2=1.09 on 1 df, p=0.3 | χ2=16.22 on 1 df, p=6e-05* |
| **1.2x10^2^CFU** | x | x | x | x | χ2=10.72 on 1 df, p=0.001 |

Table 2: The results of pairwise likelihood ratio testing using Cox Proportional Hazard model on GFP expression time-to-event data from the *Galleria* waxworms challenged with injected *Arsenophonus apicola* pOM1::GFP at different doses.

|  | **1.2x10^5^CFU** | **1.2x10^4^CFU** | **1.2x10^3^CFU** | **1.2x10^2^CFU** | **BHI control** |
| --- | --- | --- | --- | --- | --- |
| **1.2x10^6^CFU** | 0.45 on 1 df, p=0.5 | 18.12 on 1 df, p=2e-05* | 16.52 on 1 df, p=5e-05* | 23.29 on 1 df, p=1e-06* | 38.84 on 1 df, p=5e-10* |
| **1.2x10^5^CFU** | x | 11.76 on 1 df, p=6e-04* | 10.44 on 1 df, p=0.001* | 15.52 on 1 df, p=8e-05* | 29.87 on 1 df, p=5e-08* |
| **1.2x10^4^CFU** | x | x | 0 on 1 df, p=1 | 0.62 on 1 df, p=0.4 | 9.03 on 1 df, p=0.003 |
| **1.2x10^3^CFU** | x | x | x | 0.62 on 1 df, p=0.4 | 9.03 on 1 df, p=0.003 |
| **1.2x10^2^CFU** | x | x | x | x | 5.82 on 1 df, p=0.02 |

Table 3: The results of pairwise likelihood ratio testing using Cox Proportional Hazard model on melanisation time-to-event data from the *Galleria* waxworms challenged with injected *Arsenophonus apicola* pOM1::GFP at different doses.

|  | **1.2x10^5^CFU** | **1.2x10^4^CFU** | **1.2x10^3^CFU** | **1.2x10^2^CFU** | **BHI control** |
| --- | --- | --- | --- | --- | --- |
| **1.2x10^6^CFU** | 1.39 on 1 df, p=0.2 | 5.87 on 1 df, p=0.02 | 4.54 on 1 df, p=0.03 | 16.48 on 1 df, p=5e-05* | 18.48 on 1 df, p=2e-05* |
| **1.2x10^5^CFU** | x | 10.72 on 1 df, p=0.001 | 9.03 on 1 df, p=0.003 | 22.51 on 1 df, p=2e-06* | 24.83 on 1 df, p=6e-07* |
| **1.2x10^4^CFU** | x | x | 0.08 on 1 df, p=0.8 | 3.67 on 1 df, p=0.06 | 4.41 on 1 df, p=0.04 |
| **1.2x10^3^CFU** | x | x | x | 4.69 on 1 df, p=0.03 | 5.44 on 1 df, p=0.02 |
| **1.2x10^2^CFU** | x | x | x | X | 0 on 1 df, p=1 |

Table 4: The results of pairwise likelihood ratio testing using Cox Proportional Hazard model on pupation time-to-event data from the *Galleria* waxworms challenged with injected *Arsenophonus apicola* pOM1::GFP at different doses.

Injected Dose Pair-wise Heterogeneity Tables

|  | **1.1x10^5^CFU** | **1.1x10^4^CFU** | **1.1x10^3^CFU** | **1.1x10^2^CFU** | **BHI control** |
| --- | --- | --- | --- | --- | --- |
| **1.1x10^6^CFU** | 0 on 1 df p=1 | 4.51 on 1 df, p=0.03 | 4.64 on 1 df, p=0.03 | 4.64 on 1 df, p=0.03 | 5.42 on 1 df, p=0.02 |
| **1.1x10^5^CFU** | x | 4.38 on 1 df, p=0.04 | 4.64 on 1 df, p=0.03 | 4.64 on 1 df, p=0.03 | 5.42 on 1 df, p=0.02 |
| **1.1x10^4^CFU** | x | x | 0 on 1 df, p=1 | 0 on 1 df, p=1 | 0.17 on 1 df, p=0.7 |
| **1.1x10^3^CFU** | x | x | x | 0 on 1 df, p=1 | 0.17 on 1 df, p=0.7 |
| **1.1x10^2^CFU** | x | x | x | x | 0.17 on 1 df, p=0.7 |

Table 5: The results of pairwise likelihood ratio testing using Cox Proportional Hazard model on survival time-to-event data from the *Galleria* waxworms challenged with force-fed *Arsenophonus apicola* pOM1::GFP at different doses.

|  | **1.1x10^5^CFU** | **1.1x10^4^CFU** | **1.1x10^3^CFU** | **1.1x10^2^CFU** | **BHI control** |
| --- | --- | --- | --- | --- | --- |
| **1.1x10^6^CFU** | 0.47 on 1 df, p=0.5 | 6.18 on 1 df, p=0.01 | 8.27 on 1 df, p=0.004 | 11.85 on 1 df, p=6e-04* | 32.64 on 1 df, p=1e-08* |
| **1.1x10^5^CFU** | x | 6.65 on 1 df, p=0.01 | 9.17 on 1 df, p=0.002* | 12.68 on 1 df, p=4e-04* | 32.64 on 1 df, p=1e-08* |
| **1.1x10^4^CFU** | x | x | 1.53 on 1 df, p=0.2 | 4.29 on 1 df, p=0.04 | 22.51 on 1 df, p=2e-06* |
| **1.1x10^3^CFU** | x | x | x | 2 on 1 df, p=0.2 | 18.22 on 1 df, p=2e-05* |
| **1.1x10^2^CFU** | x | x | x | x | 12.48 on 1 df, p=4e-04* |

Table 6: The results of pairwise likelihood ratio testing using Cox Proportional Hazard model on GFP expression time-to-event data from the *Galleria* waxworms challenged with force-fed *Arsenophonus apicola* pOM1::GFP at different doses.

|  | **1.1x10^5^CFU** | **1.1x10^4^CFU** | **1.1x10^3^CFU** | **1.1x10^2^CFU** | **BHI control** |
| --- | --- | --- | --- | --- | --- |
| **1.1x10^6^CFU** | 0.05 on 1 df, p=0.8 | 3.11 on 1 df, p=0.08 | 6.19 on 1 df, p=0.01 | 10.94 on 1 df, p=9e-04* | 17.12 on 1 df, p=4e-05* |
| **1.1x10^5^CFU** | x | 2.6 on 1 df, p=0.1 | 5.69 on 1 df, p=0.02 | 10.74 on 1 df, p=0.001* | 16.66 on 1 df, p=4e-05* |
| **1.1x10^4^CFU** | x | x | 0.84 on 1 df, p=0.4 | 3.92 on 1 df, p=0.05 | 8.48 on 1 df, p=0.004 |
| **1.1x10^3^CFU** | x | x | x | 1.12 on 1 df, p=0.3 | 4.38 on 1 df, p=0.04 |
| **1.1x10^2^CFU** | x | x | x | x | 1.32 on 1 df, p=0.3 |

Table 7: The results of pairwise likelihood ratio testing using Cox Proportional Hazard model on melanisation time-to-event data from the *Galleria* waxworms challenged with force-fed *Arsenophonus apicola* pOM1::GFP at different doses.

|  | **1.1x10^5^CFU** | **1.1x10^4^CFU** | **1.1x10^3^CFU** | **1.1x10^2^CFU** | **BHI control** |
| --- | --- | --- | --- | --- | --- |
| **1.1x10^6^CFU** | 0.62 on 1 df, p=0.4 | 2.76 on 1 df, p=0.1 | 1.72 on 1 df, p=0.2 | 2.58 on 1 df, p=0.1 | 6.58 on 1 df, p=0.01 |
| **1.1x10^5^CFU** | x | 0.73 on 1 df, p=0.4 | 0.26 on 1 df, p=0.6 | 0.61 on 1 df, p=0.4 | 3.01 on 1 df, p=0.08 |
| **1.1x10^4^CFU** | x | x | 0.14 on 1 df, p=0.7 | 0.01 on 1 df, p=0.9 | 0.76 on 1 df, p=0.4 |
| **1.1x10^3^CFU** | x | x | x | 0.07 on 1 df, p=0.8 | 1.59 on 1 df, p=0.2 |
| **1.1x10^2^CFU** | x | x | x | x | 1.04 on 1 df, p=0.3 |

Table 8: The results of pairwise likelihood ratio testing using Cox Proportional Hazard model on pupation time-to-event data from the *Galleria* waxworms challenged with force-fed *Arsenophonus apicola* pOM1::GFP at different doses.
