# Supplementary material for: The Honeybee Associate Galleria mellonella Can Acquire Arsenophonus apicola Through Oral and Parenteral Infection Routes
Source: Environ Microbiol. 2025 Apr 13;27(4):e70088. doi: 10.1111/1462-2920.70088 (PMC11994876; doi:10.1111/1462-2920.70088)
Supplement: Supplementary file 4 — Data S4. [file EMI-27-e70088-s003.docx]

***Melanisation and Fluorescence Index Photos***

Melanisation was measured against the following index (adapted from [Kay et al, 2019]) during daily observation of larvae in the infected and control cohorts.

| Melanisation Score | Description | Example |
| --- | --- | --- |
| 0 | Unmelanised | 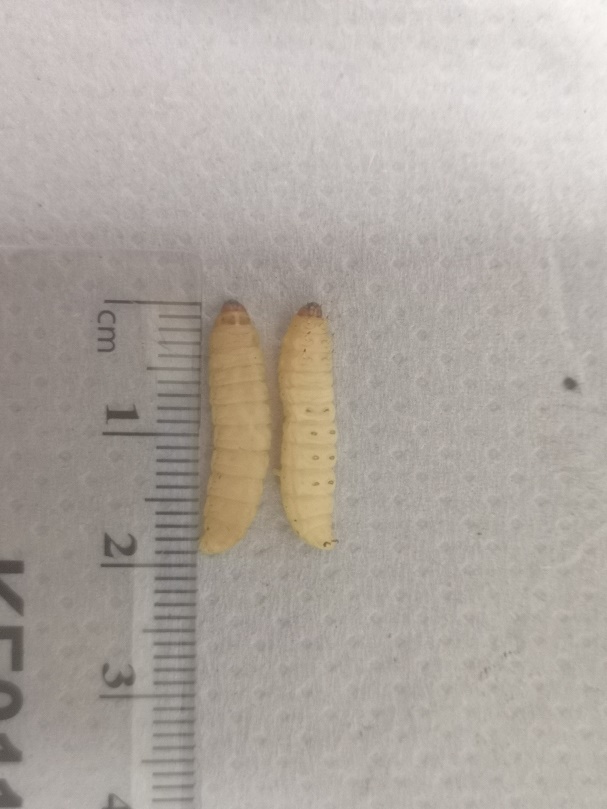 |
| 1 | Evidence of nodulation | 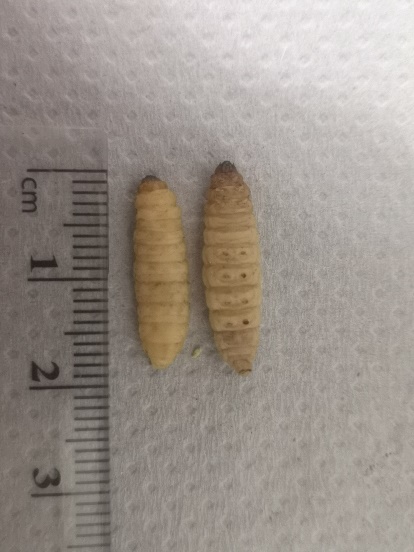 |
| 2 | “Lateral line” melanisation | 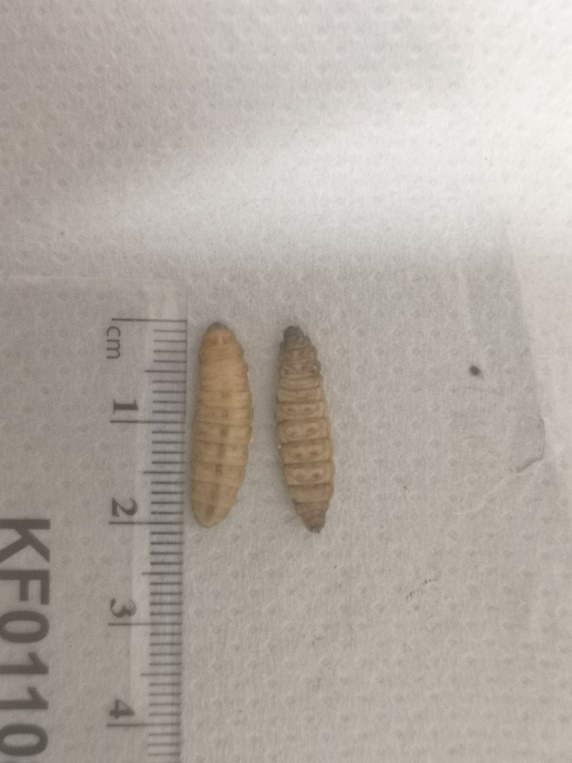 |
| 3 | Systemic melanisation (>50%) | 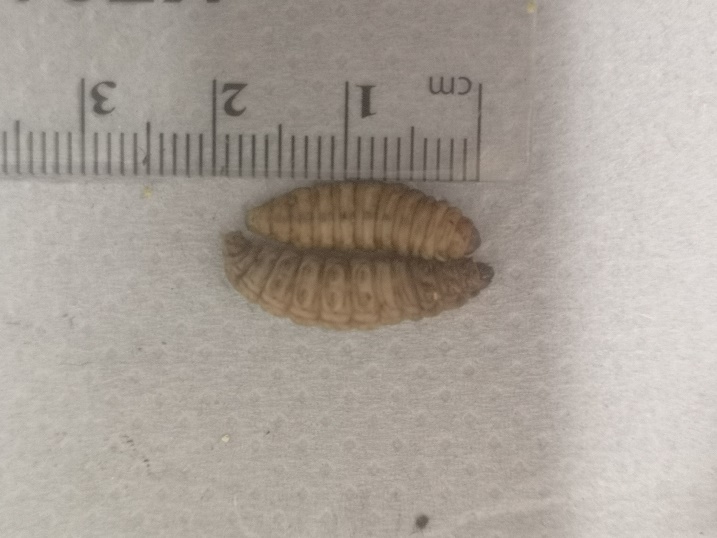 |
| 4 | Complete melanisation | 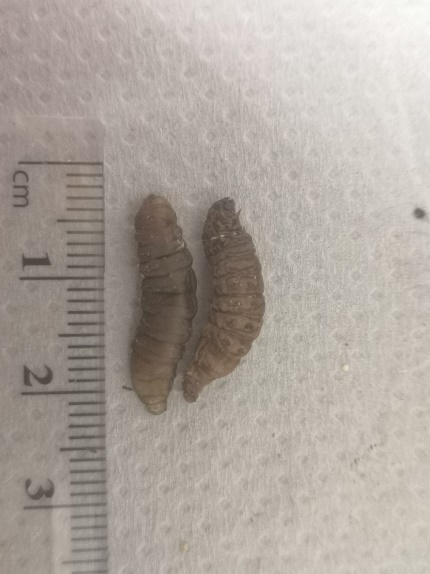 |

Fluorescence was assessed using a M165 FC Leica stereoscope equipped with a Leica EL6000 external light source for fluorescence excitation and visualized using the GFP plus filter [480/40 nm (460–500 nm); Leica Microsystems (UK) limited] during daily observation of larvae in the infected and control cohorts. Pictured below are examples of each status.

| Fluorescent Status | Example |
| --- | --- |
| Absent (Autofluorescence) | 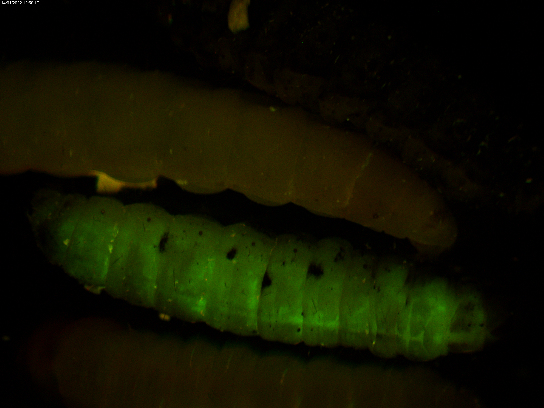 |
| Present (GFP expression) | 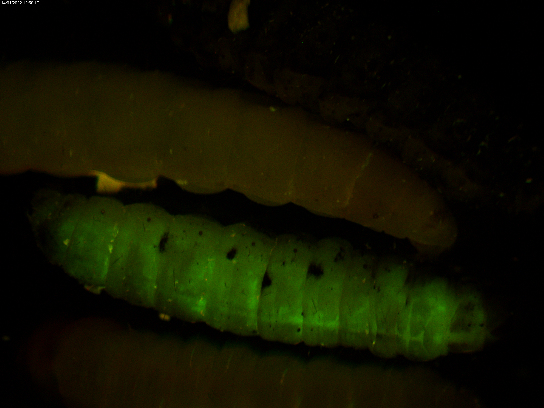 |

*References*

Kay, S., Edwards, J., Brown, J., & Dixon, R. (2019). Galleria mellonella Infection Model Identifies Both High and Low Lethality of Clostridium perfringens Toxigenic Strains and Their Response to Antimicrobials. Frontiers in Microbiology, 10, 1281. https://doi.org/10.3389/fmicb.2019.01281
